# Unveiling the Resistome Landscape in Peri-Implant Health and Disease

**DOI:** 10.3390/jcm14030931

**Published:** 2025-01-31

**Authors:** Lucinda J. Bessa, Conceição Egas, João Botelho, Vanessa Machado, Gil Alcoforado, José João Mendes, Ricardo Alves

**Affiliations:** 1 Egas Moniz Center for Interdisciplinary Research (CiiEM), Egas Moniz School of Health & Science, 2829-511 Caparica, Almada, Portugal; jbotelho@egasmoniz.edu.pt (J.B.); vmachado@egasmoniz.edu.pt (V.M.); galcoforado@egasmoniz.edu.pt (G.A.); jmendes@egasmoniz.edu.pt (J.J.M.); ralves@egasmoniz.edu.pt (R.A.); 2 CNC-UC—Center for Neuroscience and Cell Biology, Polo I, University of Coimbra, Rua Larga, Edifício FMUC, Piso 1, 3004-504 Coimbra, Portugal; conceicao.egas@biocantassociacao.pt; 3 CIBB—Center for Innovative Biomedicine and Biotechnology, University of Coimbra, Rua Larga, Edifício FMUC, Piso 1, 3004-504 Coimbra, Portugal; 4 Genoinseq—Next Generation Sequencing Unit, Biocant, BiocantPark, Núcleo 04, Lote 8, 3060-222 Cantanhede, Portugal

**Keywords:** antibiotic resistance genes (ARGs), peri-implantitis, oral metagenomes, oral resistome, saliva, subgingival peri-implant biofilm

## Abstract

**Background:** The human oral microbiome is a critical reservoir for antibiotic resistance; however, subgingival peri-implant biofilms remain underexplored in this context. We aimed to explore the prevalence and distribution of antibiotic resistance genes (ARGs) in metagenomes derived from saliva and subgingival peri-implant biofilms. **Methods:** A total of 100 metagenome datasets from 40 individuals were retrieved from the Sequence Read Archive (SRA) database. Of these, 20 individuals had exclusively healthy implants and 20 had both healthy and affected implants with peri-implantitis. ARGs and their taxonomic assignments were identified using the ABRicate tool, and plasmid detection was performed with PlasmidFinder. **Results:** Four plasmid replicons were identified in 72 metagenomes, and 55 distinct ARGs from 13 antibiotic classes were detected in 89 metagenomes. ARGs conferring resistance to macrolides–lincosamides–streptogramins, tetracyclines, beta-lactams, and fluoroquinolones were the most prevalent. The *msr*(D) and *mef*(A) genes showed the highest prevalence, except in saliva samples from individuals with healthy implants, where *mef*(A) ranked fourth. A pairwise PERMANOVA of principal coordinate analysis based on Jaccard distances revealed that saliva samples exhibited significantly greater ARG diversity than subgingival biofilm samples (*p* < 0.05). However, no significant differences were observed between healthy and peri-implantitis-affected subgingival biofilm groups (*p* > 0.05). The taxonomic origins of ARGs were also analyzed to understand their distribution and potential impact on oral microbial communities. **Conclusions:** Resistome profiles associated with both peri-implant health and disease showed no significant differences and higher salivary abundance of ARGs compared to subgingival biofilm samples.

## 1. Introduction

The oral microbiome is among the most diverse in the human body and plays a critical role in maintaining oral health. The microbial community composing the oral microbiome is typically arranged in biofilms. Oral biofilms form not only on natural teeth but also on restorative materials, dental prosthetic devices, and dental implants [1,2]. Bacteria growing within a biofilm often exhibit altered phenotypes, being more tolerant to antimicrobial agents, host immune defense mechanisms, and mechanical removal [3]. The structural properties and proximity of the bacterial cells within the biofilm facilitate horizontal gene transfer, which can lead to the spread of antibiotic resistance genes (ARGs) [1,4]. Furthermore, numerous ARGs are found on mobile genetic elements (MGEs), such as transposons and conjugative elements (e.g., plasmids), facilitating their efficient transfer between cells, including from non-pathogenic and commensal bacteria to pathogenic species [5].

The collection of ARGs present in the microbiome of the oral cavity is referred to as the oral resistome [6,7,8,9]. A comprehensive evaluation of ARG distribution in specific oral niches is essential for understanding the oral resistome, though its overall complexity remains poorly understood [10,11]. Similarly to the oral microbiome, the oral resistome is highly individualized [12], limiting its general knowledge. Advances in genomic technologies, particularly next-generation sequencing (NGS), are transforming our understanding of the microbiome and driving the emergence of microbiome medicine, which aims to leverage human microbiota and their derived molecules for the prevention and treatment of diseases [13]. These tools are also essential for decoding the oral resistome and its role in antimicrobial resistance (AMR) dissemination [11].

Dental implants have revolutionized oral rehabilitation, yet peri-implantitis—a biofilm-associated inflammatory disease affecting the tissues around implants—remains a major clinical challenge. Peri-implantitis can lead to progressive bone loss and, ultimately, implant failure if left untreated. Peri-implantitis is estimated to affect around 20% of individuals with dental implants [14], highlighting its widespread prevalence. The clinical management of PI is complicated by the complex biofilm composition, which can render conventional treatments, such as mechanical debridement and antibiotic therapy, less effective [15,16,17]. Understanding the microbial and resistome profiles of peri-implant biofilms is therefore essential to developing more effective therapeutic strategies.

In clinical dental practice, antibiotics are frequently prescribed to treat oral infections or prophylactically to prevent systemic and local complications associated with dental procedures [6,18]. However, these prescriptions are often based on empirical clinical judgment rather than definitive diagnoses, contributing to the growing concern over inappropriate antibiotic use in dentistry and the consequent pressing need for dental antibiotic stewardship [18,19]. This study aimed to (i) identify ARGs and plasmids within metagenomic datasets derived from saliva and subgingival peri-implant biofilm samples collected from patients with healthy implants and those with a mix of peri-implantitis-affected implants and healthy implants and (ii) attribute taxonomic origins to the detected ARGs.

A total of 55 distinct ARGs conferring resistance to 13 antibiotic classes were identified across 89 of the 100 metagenomes. While ARG profiling revealed no significant differences between healthy and peri-implantitis-affected implants, salivary samples exhibited a higher prevalence of ARGs compared to subgingival biofilm samples.

## 2. Materials and Methods

### 2.1. Data Acquisition and Information

A total of 100 metagenome datasets were downloaded from the NCBI SRA database (https://www.ncbi.nlm.nih.gov/sra (accessed on 30 September 2024)) under NCBI BioProject number PRJNA1163384 and used in this study (https://www.ncbi.nlm.nih.gov/sra/?term=PRJNA1163384 (accessed on 30 September 2024)). The datasets were derived from 40 individuals from Portugal with one or more dental implants. Of these, 20 individuals had exclusively healthy implants (HI group), while the remaining 20 had a co-occurrence of healthy implants and implants affected by peri-implantitis (PI group). Each participant provided a saliva sample and one (in the HI group) or two (in the PI group) subgingival biofilm samples. Each biosample or metagenome was categorized into one of five study groups, namely HI_Sa, HI_HIS, PI_Sa, PI_HIS, and PI_PIS. The abbreviations are as follows: “Sa” for saliva, “HIS” for healthy implant site, and “PIS” for peri-implantitis-affected site. Detailed information, including accession numbers, is provided in Table S1 in the Supplementary Materials.

### 2.2. Data Analysis and Statistics

Sequenced reads were quality-filtered with Trimmomatic version 0.39 [20] using the following parameters: (1) sequencing adapters were removed, (2) bases with an average quality lower than Q25 in a window of five bases were trimmed, and (3) reads with less than 100 bases were discarded. High-quality reads were filtered against the reference human genome sequence assembly GRCh38/hg38 with Bowtie version 2.5 [21]. High-quality sequences from each sample were de novo assembled with metaSPAdes version 3.15.5 [22], using default parameters. Metagenome-assembled contigs were analyzed for the presence of ARGs with ABRicate version 1.0.1 [23] with the ResFinder [24], CARD [25], NCBI [26], and ARG-ANNOT [27] databases. ARGs were identified with a sequence identity of at least 90% and coverage of at least 80%. A taxonomic classification of genes was provided for each database result. ARGs were classified into antibiotic resistance classes according to the CARD database and literature searches. The taxonomic attribution of each identified ARG was retrieved directly from the ABRicate output results or from the GenBank accession numbers also present in the ABRicate outputs. Metagenome-assembled contigs were also analyzed for the presence of plasmids with PlasmidFinder within the staramr package version 0.10 [28]. Plasmids were searched against the complete PlasmidFinder database version 2.2 [29]. Plasmids were identified with a sequence identity of at least 90% and coverage of at least 80%. The plasmidome was characterized through prevalence analysis per study group.

The resistome was characterized through prevalence analysis at the gene and antibiotic class level per study group. Differences between group prevalences were analyzed by principal coordinate analysis (PCoA) in phyloseq version 1.44.0 [30] using the Jaccard similarity coefficient. The indexes were tested for statistical differences with PERMANOVA, followed by pairwise PERMANOVA using the adonis function of the vegan package version 2.6-4 [31] with 1000 permutations and the Benjamani–Hochberg procedure for multiple comparison corrections. Homoscedasticity was tested with the betadisper function of the vegan package. The differential prevalence of genes between study groups was further analyzed by Firth’s logistic regression with the logistf package, version 1.26.0 [32]. Multiple comparisons were corrected using the Benjamani–Hochberg procedure.

A Circos representation of the prevalence of antibiotic classes and study groups was created on the online version of the tool [33].

Beta diversity (Jaccard distance), prevalence, and Firth’s logistic regression analysis were performed using R Statistical Software version 4.4.1 [34] in RStudio version 2024.09.0 build 375 [35]. Plots were produced with ggplot2 version 3.5.1 [36]. A *p*-value and an adjusted *p*-value of < 0.05 were considered statistically significant.

## 3. Results

### 3.1. Detection and Prevalence of ARGs and Plasmids Across Study Groups

ARGs were detected in 89 out of the 100 samples, while plasmids were identified in 72. Samples with no detectable ARGs or plasmids are listed in the Supplementary Materials, Tables S2 and S3, respectively.

Four kinds of plasmids were identified in the samples. The most prevalent was repUS43, followed by repUS38 and repUS34. The repUS37 plasmid replicon was only found in one sample of group HI_Sa (Figure S1 in the Supplementary Materials). These plasmids typically harbor multiple ARGs; for instance, the repUS43 plasmid encodes both *tet(M)* and *erm(B)*, which confer resistance to tetracyclines and macrolides–lincosamides–streptogramins (MLSs), respectively [37]. This genetic composition significantly contributes to multidrug resistance and its spread among bacterial populations.

Overall, 55 different ARGs conferring resistance to 13 antibiotic classes were found. The ARGs and corresponding specific antibiotic classes are listed in Table S4 in the Supplementary Materials. All 89 samples with ARGs harbored multiple distinct ARGs.

**Figure 1:**
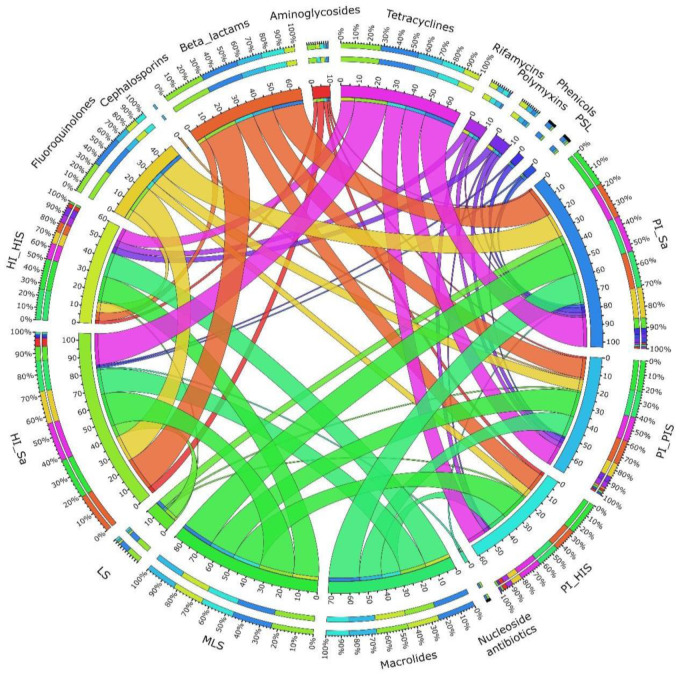
Circos diagram illustrating the prevalence of antibiotic classes and their associations with the respective study groups. LS: lincosamide–macrolide; MLS: macrolide–lincosamide–streptogramin; PLS: pleuromutilin–streptogramin–lincosamide.

ARGs conferring resistance to MLS, macrolides, tetracyclines, beta-lactams, and fluoroquinolones were most prevalent in all study groups, particularly in saliva samples (HI_Sa and PI_Sa groups) as shown in Figure 1 and Figures S2 and S3 in the Supplementary Materials. ARGs conveying resistance to pleuromutilins–streptogramins–lincosamides (PSLs), phenicols, nucleoside antibiotics, and cephalosporins were less prevalent across all study groups. Notably, aminoglycoside resistance genes were particularly less prevalent in the PI_Sa and PI_PIS groups (Figure 1 and Figure S3 in the Supplementary Materials).

**Figure 2:**
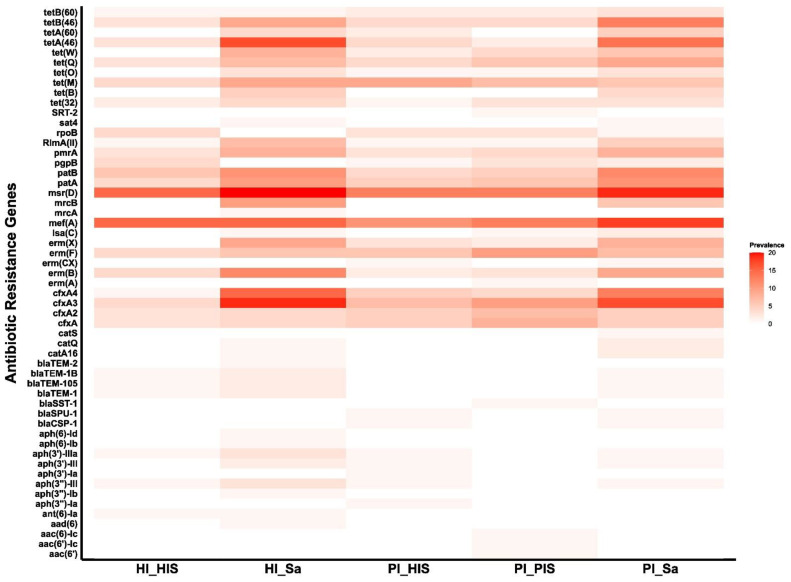
Heatmap showing the prevalence of ARGs across each of the five study groups, PI_Sa, PI_HIS, PI_PIS, HI_Sa, and HI_HIS.

The prevalence of ARGs per study group is shown in Figure 2 and Figure S4 in the Supplementary Materials. Among the total 55 ARGs identified, 19 were shared across all study groups.

The *msr(D)* and *mef(A)* genes, conferring resistance to MLS and macrolides, respectively, ranked highest in prevalence across all study groups, except in the HI_Sa group, where *mef(A)* was the fourth most common.

A PCoA based on the Jaccard distance was performed to evaluate differences in group prevalences (Figure S5, Supplementary Materials), and PERMANOVA analysis indicated significant overall differences (*p* = 0.001). Pairwise PERMANOVA showed that saliva groups (HI_Sa and PI_Sa) harbored a significantly higher number of ARGs than the subgingival peri-implant biofilm groups (PI_HIS, PI_PIS, and HI_HIS), with *p* < 0.05 (Table S5 in the Supplementary Materials). However, though peri-implantitis-affected implants (PI_PIS group) generally displayed a higher prevalence of most ARGs than healthy implants (PI_HIS and HI_HIS groups), the difference was not statistically significant (*p* > 0.05).

**Figure 3:**
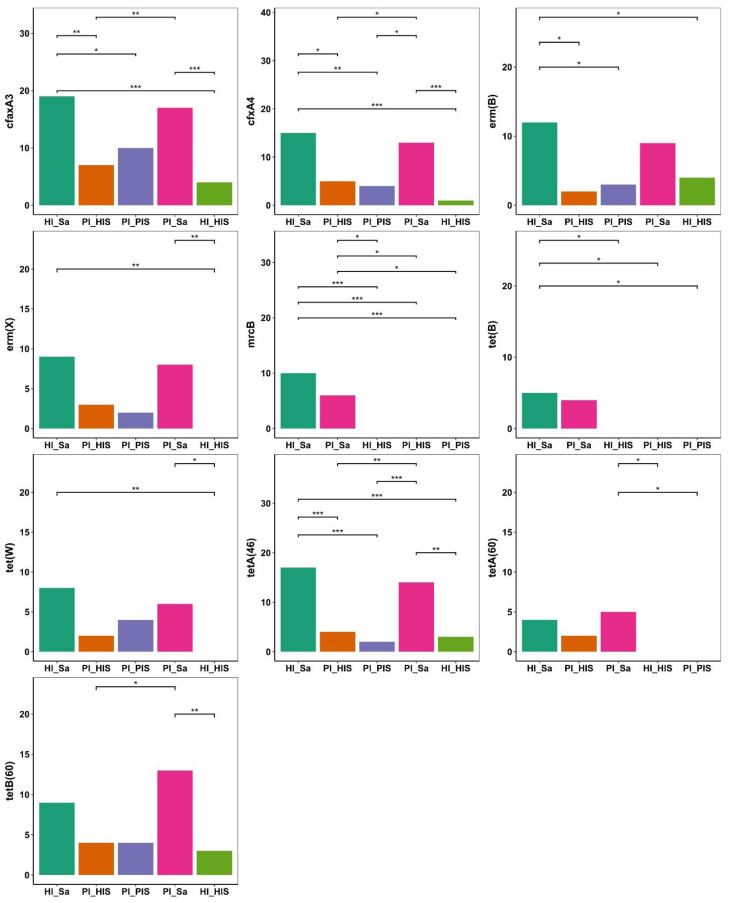
Differentially prevalent ARGs between the study groups, with significance determined by Firth’s logistic regression * *p* ≤ 0.05, ** *p* ≤ 0.01, and *** *p* ≤ 0.001.

Ten ARGs were differentially prevalent among study groups (Figure 3).

The genes *tet(W)*, *erm(X)*, *mrcB*, and *tetA(60)* were detected in the HI_Sa group but were absent in the HI_HIS group. The genes *tet(B)* and *mrcB* were only detected in saliva groups.

### 3.2. Taxonomic Assignment of Identified ARGs

**Figure 4:**
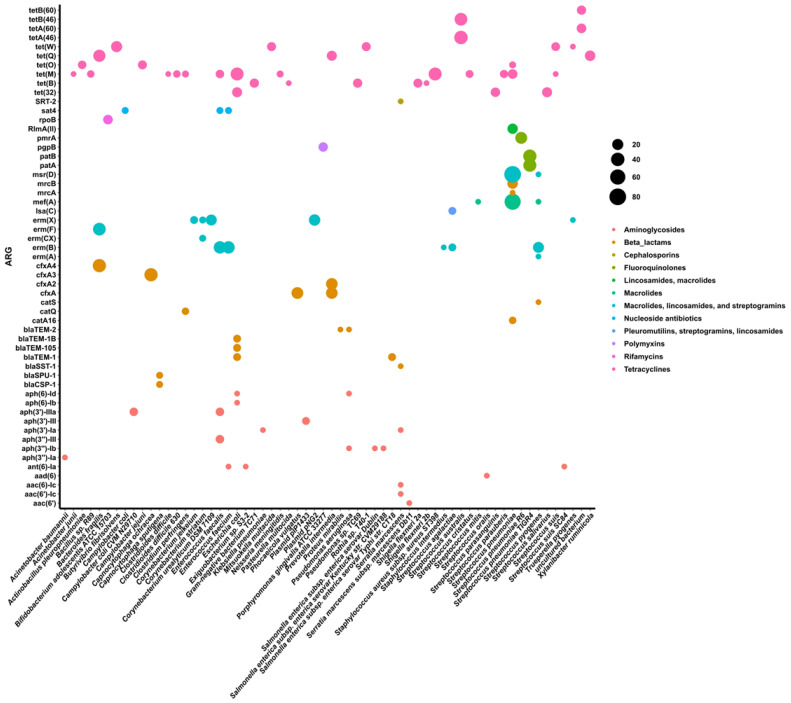
Distribution of ARGs per bacterial species/plasmid. The size of the dots represents the prevalence of each gene within the respective bacterial species or plasmid. The taxonomic attribution of each identified ARG was retrieved directly from the ABRicate.

To estimate the taxonomy of ARGs, the taxonomic attribution for each identified ARG was directly retrieved from ABRicate. Figure 4 displays each ARG along with its corresponding bacterial species and its prevalence within each species across all samples, regardless of the study group.

Several distinct ARGs were associated with individual bacterial species. For example, *Escherichia coli* was linked to *tet(M)*, *tet(32)*, *bla*_TEM-1_, *bla*_TEM-1B_, *bla*_TEM-105_, *aph*(6)*-Id*, and *aph*(6)*-Ib*; *Enterococcus faecalis* was associated with *tet(M)*, *sat4*, *erm(B)*, *aph*(3′)*-IIIa*, and *aph*(3″)-*III*; and *Streptococcus pneumoniae* with *tet(O)*, *tet(M)*, *RlmA(II)*, *msr(D)*, *mef(A)*, *mrcB*, *mrcA*, and *catA16*. Some ARGs were also specifically associated with oral bacterial species, including *Porphyromonas gingivalis* (*pgpB*), *Prevotella intermedia* (*tet(Q)*, *cfxA*, *cfxA2*), and *Streptococcus salivarius* and *S. parasanguinis* (*tet(32)*). Remarkably, the *pgpB*, *cfxA*, and *cfxA2* genes were detected across all study groups, as shown in Figure 2.

Genes encoding resistance to tetracyclines stand out due to their widespread presence across a diverse range of bacterial species (Figure 4). The gene *tet(M)* was assigned to 13 different species.

The *erm(X)* gene was predominantly associated with *Corynebacterium urealyticum* and the *Corynebacterium diphtheriae* plasmid, the plasmid pNG2. Additionally, the *aph(3*′)-*III* gene, encoding resistance toward aminoglycosides, was also identified within a plasmid, the plasmid plP1433.

## 4. Discussion

In this secondary study, metagenomes from saliva and subgingival peri-implant biofilm samples were explored for the presence of ARGs and plasmids. This first metagenomic sequencing study reports that implants affected by peri-implantitis generally exhibited a higher prevalence of most ARGs compared to healthy implants but were not statistically significant. However, dental implants were confirmed as reservoirs for ARGs and plasmids. We also confirmed that salivary samples present higher levels of ARGs and plasmids compared to subgingival samples.

To gain insights into the ARG profiles of these samples, we integrated multiple databases (including ABRicate, CARD, ResFinder, NCBI, and ARG-ANNOT), a common procedure to enhance the comprehensiveness and accuracy of ARG detection [13]. We used the PlasmidFinder database to identify plasmid replicons in the metagenomes [29].

Three plasmid replicons (repUS43, repUS38, and repUS34) were found in all study groups; however, they were slightly more prevalent in salivary than in subgingival peri-implant samples. The repUS43 replicon is a conjugative plasmid frequently identified in *Enterococcus* spp. isolates [38] and is associated with ARGs, including *tet(M)* and *erm(B)* [32]. Conjugative plasmids are self-transmissible, enabling their transfer between bacterial cells through conjugation. Consequently, plasmids like repUS43, which carry multiple ARGs, may play a pivotal role in disseminating antibiotic resistance, particularly in these bacterial communities of the oral cavity. Their presence in this niche facilitates horizontal gene transfer among resident and pathogenic bacteria, further complicating treatment strategies and posing a significant challenge to the clinical management of oral and systemic infections.

The metagenomic analysis revealed a high prevalence of antibiotic resistance genes (ARGs) across all five study groups. In total, ARGs conferring resistance to 13 antibiotic classes were identified, with MLS, macrolides, tetracyclines, beta-lactams, and fluoroquinolones being the most prevalent. These antibiotic classes are ranked by the World Health Organization (WHO) as critically or highly important for human medicine [39]. The most commonly detected ARGs were associated with mechanisms such as antibiotic efflux (e.g., *mef(A)*, *msr(D)*, *patA*, and *patB*), antibiotic inactivation via beta-lactamases (*cfxA*, *cfxA3*), and inhibition of protein synthesis (*tet(M)* and *erm(F)*). The *msr(D)* and *mef(A)* genes were consistently the most prevalent. Notably, it has been reported that in mef-carrying genetic elements, the *msr(D)* gene is invariably associated with and co-transcribed with the *mef(A)* gene, playing a key role in macrolide efflux resistance [40,41,42]. The *mef*(A) and *msr(D)* genes form a two-component ATP-binding cassette (ABC) efflux transport system, with *mef(A)* encoding the transmembrane channel and *msr(D)* encoding the two ATP-binding domains. However, in the absence of Mef(A), Msr(D) likely utilizes an alternative transmembrane channel for macrolide efflux [40].

The analyzed metagenomes were obtained from samples collected from individuals in Portugal. A recent cross-sectional web survey revealed that 64% of Portuguese dentists consistently use antibiotic prophylaxis for dental implant placement, 29% prescribe antibiotics postoperatively, and 55% use both pre- and postoperative regimens. Amoxicillin combined with clavulanic acid was the most prescribed antibiotic (57%) [43]. Similarly, a questionnaire-based study investigating European dentists’ prescribing practices for dental implant treatments found that amoxicillin alone (61.0%) or combined with clavulanic acid (55.9%) were the most commonly prescribed antibiotics, followed by clindamycin (32.3%), erythromycin, and azithromycin [44]. These findings highlight a high rate of antibiotic prescriptions for dental implant procedures. Such overprescription contributes significantly to the rise in antibiotic resistance and the prevalence of antibiotic resistance genes (ARGs) in both healthy and diseased oral environments.

To our knowledge, this is the first study to report the distribution patterns of ARGs in both healthy implants and those affected by peri-implantitis based on shotgun metagenomic sequencing data. Nevertheless, similar metagenomic studies have been conducted on dental plaque from periodontal healthy and diseased sites to characterize its resistome [7,9,45]. Kang et al. [7] reported that resistance genes for bacitracin, beta-lactams, MLS, and tetracyclines were present in almost all dental plaque samples at relatively high abundances. However, periodontitis was associated with an increased number of ARGs and significant alterations in the composition of ARGs in dental plaque compared to the healthy condition [7]. In another study, Anderson et al. [9] identified *mef(A)*, *msr(D)*, *cfxA*, and *erm(F)* as the most prevalent ARGs in healthy oral biofilm, whereas *tet(Q)*, *pgpB*, and *tet(32)* were more common in cases of periodontitis. Gager et al. [45] examined the prevalence of ARGs in the subgingival biofilm of a population of German patients diagnosed with periodontitis. Their study revealed that macrolide resistance genes were the most prevalent and that two-thirds of the participants carried ARGs associated with at least one class of antibiotics commonly used in periodontal treatment and research, including beta-lactams, lincosamides, macrolides, nitroimidazoles, and tetracyclines.

A total of 40 different ARGs were identified in saliva samples from both the HI_Sa and PI_Sa groups, representing a higher number than those detected in the subgingival peri-implant biofilm groups (26 in HI_HIS, 32 in PI_HIS, and 30 in PI_PIS). The genes *tet(B)* and *mrcB* were detected in saliva groups but not in the subgingival biofilm groups. In both saliva groups, the majority of ARGs, including the most prevalent ones, were shared between the two groups. Regarding antibiotic classes, the order of prevalence in the HI_Sa group was MLS, beta-lactams, tetracyclines, fluoroquinolones, and macrolides. In the PI_Sa group, the order was MLS, tetracyclines, macrolides, beta-lactams, and fluoroquinolones. Similarly, Caselli et al. [46], using a microarray approach to analyze the oral resistome in oral rinse samples (not saliva) from healthy Italians, also reported a high prevalence of resistance to MLS and tetracyclines.

The *tet(M)* gene was the most prevalent tetracycline resistance gene in all three subgingival peri-implant biofilm groups, while in the saliva groups, *tetA(46)* was predominant. Similarly, Anderson et al. [9] reported that *tet(M)* was the most abundant tetracycline resistance gene in healthy dental plaque samples. Kang et al. [7] also observed a high prevalence of *tet(M)* in dental plaque samples both with and without periodontitis. Notably, the *tet(M)* gene is often linked to the *erm(B)* gene, as both are frequently found on conjugative plasmids or transposons, such as repUS43 and Tn916 [8]. This association suggests that the abundance of tetracycline resistance genes in the dental plaque resistome may result from co-selection pressures. The use of erythromycin, for example, can co-select for tetracycline resistance, which could explain the high prevalence of *tet(M)* in oral metagenomes [7,8]. Consistent with this, the *tet* genes identified in our studied metagenomes were widely distributed and taxonomically associated with various bacterial species, encompassing both Gram-positive and Gram-negative bacteria. Particularly, the *tet(M*) gene was assigned to 13 different bacterial species.

Based on the taxonomic attribution of each identified ARG, we identified 62 bacterial species and two plasmids as potential hosts for 55 ARGs. We refer to them as potential hosts because the taxonomic assignment obtained should be considered indicative rather than definitive, given the challenges associated with assigning ARGs to specific taxa. These challenges include (i)) ARGs are frequently transferred between taxa via mechanisms of horizontal gene transfer; this mobility complicates taxonomic assignment, as the ARG may not belong to the taxon indicated by nearby genomic markers, (ii) short reads and fragmented assemblies can separate ARGs from their surrounding genomic context, making it difficult to establish a reliable taxonomic link [47], and (iii) reference databases for ARGs and taxonomy may be incomplete or biased toward well-studied organisms, leading to potential misassignments or missed identifications [48]. Acknowledging these limitations, our goal was to obtain an approximate understanding of the distribution of ARGs across different species.

The taxonomic assignment of ARGs revealed that typical commensal taxa, such as *Rothia* spp. and *Bifidobacterium* spp., were associated with resistance genes for tetracyclines and rifamycins, respectively. Gram-positive bacteria, including *Streptococcus* spp., *Enterococcus* spp., *Corynebacterium* spp., and *Clostridioides* spp., carried a diverse array of ARGs conferring resistance to MLS and tetracyclines. Additionally, ARGs conferring resistance to macrolides, lincosamides, and fluoroquinolones were attributed to *Streptococcus pneumoniae*. Among Gram-negative bacteria, *Escherichia coli* and *Prevotella intermedia* predominantly harbored ARGs for beta-lactam and tetracycline resistance. Also consistent with other research, the production of β-lactamase in *Prevotella* species has been linked to the expression of the *cfxA* and *cfxA2* genes [49,50]. Additionally, resistance to polymyxins was exclusively associated with *Porphyromonas gingivalis*. In a previous study [9], *P. gingivalis* was also associated with the *pgpB* gene, which encodes resistance to peptide antibiotics such as polymyxins.

Two genes were taxonomically assigned to plasmids. The *erm(X)* gene was predominantly associated with the plasmid pNG2, which is known to mediate inducible resistance to erythromycin and other MLS antibiotics [51]. The *aph(3*′)-*III* gene had previously been identified within the plasmid plP1433 [52].

These putative ARG-carrying pathogens have the potential to compromise the efficacy of commonly used antibiotics, highlighting the need for targeted antimicrobial strategies and the judicious use of antibiotics in dental implantology. It is important to note, however, that bacteria may harbor an ARG that is not expressed, meaning it would not result in phenotypic resistance [53]. Nonetheless, Anderson et al. [9] reported a notably high concordance of nearly 60% between genotypic and phenotypic analyses. Furthermore, the same study highlighted that while metagenomic analysis has greatly advanced our understanding of the distribution and diversity of ARGs within the microbiome, it has limitations. Specifically, current bioinformatic methods are often unable to detect point mutations, which means resistance to certain antibiotics, such as vancomycin, may go undetected at the genomic level despite being phenotypically present [9]. This limitation also applies to our study, as we used the ABRicate tool, which is designed to detect acquired resistance genes but is not capable of identifying point mutations [9].

Another limitation is the sample size in our current dataset, which prevented us from conducting further analyses to explore potential correlations between ARGs and the severity of peri-implantitis or prognostic predictions for dental implants. Our primary concern was that performing such statistical analyses might lead to overfitting and yield unreliable conclusions. However, we acknowledge the significance of such analyses and aim to address these questions in future studies with larger sample sizes, which will facilitate more robust statistical evaluations and clinical insights.

Dental implants can serve as significant reservoirs for biofilm-associated ARGs, posing potential risks not only to oral health but also to systemic health. Biofilms formed on dental implants provide a protective environment for ARG-carrying bacteria, enabling their persistence, horizontal gene transfer, and potential dissemination. Similarly to findings in periodontitis, where periodontal bacteria have been implicated in complications such as ventilator-associated pneumonia due to their migration to the respiratory tract, implant-associated biofilms may contribute to analogous risks [54,55,56,57]. The ability of peri-implant bacteria to enter systemic circulation or adjacent anatomical regions underscores the importance of understanding and mitigating the role of dental implants as reservoirs of ARGs. Further studies are required to elucidate the extent to which implant-associated biofilms contribute to systemic antibiotic resistance and related health complications.

## 5. Conclusions

Comparable levels of ARGs were observed between healthy and peri-implantitis subgingival samples, as well as between salivary samples. The prevalence of ARGs was higher in salivary samples compared to subgingival peri-implant biofilms. The most prevalent ARGs were those conferring resistance to macrolides–lincosamides–streptogramins, tetracyclines, beta-lactams, and fluoroquinolones. Future studies should explore the functional impact of these ARGs and clinical relevance in dental and medical settings.

## Data Availability

The metagenomic data used in this study are available in the NCBI SRA database (https://www.ncbi.nlm.nih.gov/sra (accessed on 30 September 2024)) under NCBI BioProject number PRJNA1163384 (https://www.ncbi.nlm.nih.gov/sra/?term=PRJNA1163384 (accessed on 30 September 2024)).

## Supplementary Materials

The following supporting information can be downloaded at: https://www.mdpi.com/article/10.3390/jcm14030931/s1, Figure S1: Prevalence of plasmids (detected using the PlasmidFinder database) across each of the five study groups (PI_HIS, PI_PIS, PI_Sa, HI_Sa and HI_HIS); Figure S2: Prevalence of ARGs by antibiotic classes across all samples in which ARGs were detected (n = 89); Figure S3: Prevalence of ARGs by antibiotic classes across each of the five study groups (PI_Sa, PI_HIS, PI_PIS, HI_Sa and HI_HIS); Figure S4: Prevalence of ARGs across each of the five study groups (PI_Sa, PI_HIS, PI_PIS, HI_Sa and HI_HIS); Figure S5: PCoA of Jaccard distance showing the dissimilarity of the ARG profiles between groups. PERMANOVA: R^2^ = 0.122, p = 0.001; Table S1: Basic information on the datasets obtained from the 100 metagenomes deposited on NCBI SRA database under the BioProject number PRJNA1163384. Origin of samples: Portugal; data type: Illumina; reads length: 150 bp; Table S2: The samples in which no ARGs were detected; Table S3: The samples in which no plasmids were detected; Table S4: ARGs and their corresponding antibiotic class assignments; Table S5: Pairwise PERMANOVA based on the Jaccard coefficient between study groups.
