# Supplementary material for: Unveiling the Resistome Landscape in Peri-Implant Health and Disease
Source: J Clin Med. 2025 Jan 31;14(3):931. doi: 10.3390/jcm14030931 (PMC11818638; doi:10.3390/jcm14030931)
Supplement: Supplementary file 1 [file jcm-14-00931-s001.zip › jcm-3429354-supplementary.pdf]

Journal of Clinical Medicine

Supplementary Materials

Table S1. Basic information on the datasets obtained from the 100 metagenomes deposited on NCBI SRA database under the BioProject number PRJNA1163384. Origin of samples: Portugal; data type: Illumina; reads length: 150 bp.

Sample	Study group	NCBI accession
4sa	HI_Sa	SRR30751046
4is	HI_HIS	SRR30751045
7sa	PI_Sa	SRR30751034
7is	PI_HIS	SRR30751023
7pi	PI_PIS	SRR30750980
10sa	HI_Sa	SRR30750969
10is	HI_HIS	SRR30750958
12sa	HI_Sa	SRR30751011
12is	HI_HIS	SRR30751000
13sa	PI_Sa	SRR30750989
13is	PI_HIS	SRR30751044
13pi	PI_PIS	SRR30751043
14sa	PI_Sa	SRR30751042
14is	PI_HIS	SRR30751041
14pi	PI_PIS	SRR30751040
18sa	PI_Sa	SRR30751039
18is	PI_HIS	SRR30751038
18pi	PI_PIS	SRR30751037
19sa	PI_Sa	SRR30751036
19is	PI_HIS	SRR30751035
19pi	PI_PIS	SRR30751033
20sa	HI_Sa	SRR30751032
20is	HI_HIS	SRR30751031
21sa	PI_Sa	SRR30751030
21is	PI_HIS	SRR30751029
21pi	PI_PIS	SRR30751028
23sa	PI_Sa	SRR30751027
23is	PI_HIS	SRR30751026
23pi	PI_PIS	SRR30751025
25sa	HI_Sa	SRR30751024
25is	HI_HIS	SRR30751022
26sa	HI_Sa	SRR30751021
26is	HI_HIS	SRR30751020
27sa	PI_Sa	SRR30751019
27is	PI_HIS	SRR30751018
27pi	PI_PIS	SRR30751017
28sa	PI_Sa	SRR30751016
28is	PI_HIS	SRR30751015

28pi	PI_PIS	SRR30750982
29sa	PI_Sa	SRR30750981
29is	PI_HIS	SRR30750979
29pi	PI_PIS	SRR30750978
31sa	PI_Sa	SRR30750977
31is	PI_HIS	SRR30750976
31pi	PI_PIS	SRR30750975
32sa	HI_Sa	SRR30750974
32is	HI_HIS	SRR30750973
33sa	HI_Sa	SRR30750972
33is	HI_HIS	SRR30750971
34sa	HI_Sa	SRR30750970
34is	HI_HIS	SRR30750968
35sa	PI_Sa	SRR30750967
35is	PI_HIS	SRR30750966
35pi	PI_PIS	SRR30750965
36sa	HI_Sa	SRR30750964
36is	HI_HIS	SRR30750963
37sa	HI_Sa	SRR30750962
37is	HI_HIS	SRR30750961
38sa	HI_Sa	SRR30750960
38is	HI_HIS	SRR30750959
39sa	HI_Sa	SRR30750957
39is	HI_HIS	SRR30750956
40sa	HI_Sa	SRR30750955
40is	HI_HIS	SRR30750954
41sa	HI_Sa	SRR30750953
41is	HI_HIS	SRR30750952
42sa	HI_Sa	SRR30750951
42is	HI_HIS	SRR30751014
43sa	HI_Sa	SRR30751013
43is	HI_HIS	SRR30751012
44sa	PI_Sa	SRR30751010
44is	PI_HIS	SRR30751009
44pi	PI_PIS	SRR30751008
45sa	PI_Sa	SRR30751007
45is	PI_HIS	SRR30751006
45pi	PI_PIS	SRR30751005
46sa	HI_Sa	SRR30751004
46is	HI_HIS	SRR30751003
47sa	PI_Sa	SRR30751002
47is	PI_HIS	SRR30751001
47pi	PI_PIS	SRR30750999
48sa	PI_Sa	SRR30750998
48is	PI_HIS	SRR30750997
48pi	PI_PIS	SRR30750996

50sa	HI_Sa	SRR30750995
50is	HI_HIS	SRR30750994
51sa	HI_Sa	SRR30750993
51is	HI_HIS	SRR30750992
52sa	PI_Sa	SRR30750991
52is	PI_HIS	SRR30750990
52pi	PI_PIS	SRR30750988
53sa	PI_Sa	SRR30750987
53is	PI_HIS	SRR30750986
53pi	PI_PIS	SRR30750985
56sa	PI_Sa	SRR30750984
56is	PI_HIS	SRR30750983
56pi	PI_PIS	SRR30750950
57sa	PI_Sa	SRR30750949
57is	PI_HIS	SRR30750948
57pi	PI_PIS	SRR30750947

Table S2. The samples in which no ARGs were detected.

Sample	Study group
19is	PI_Sa
23sa	PI_PIS
31pi	PI_HIS
32is	HI_Sa
35is	PI_Sa
43is	HI_Sa
45is	PI_Sa
45pi	PI_HIS
4is	HI_Sa
52pi	PI_HIS
57is	PI_Sa

Table S3. The samples in which no plasmids were detected.

Sample	Study group
4is	HI_HIS
4sa	HI_Sa
13pi	PI_PIS
14is	PI_HIS
19is	PI_HIS
19pi	PI_PIS
21pi	PI_PIS
21sa	PI_Sa
25sa	HI_Sa
26is	HI_HIS
27pi	PI_PIS

28pi	PI_PIS
31is	PI_HIS
31pi	PI_PIS
32is	HI_HIS
35is	PI_HIS
37is	HI_HIS
40sa	HI_Sa
44pi	PI_PIS
44sa	PI_Sa
45is	PI_HIS
45pi	PI_PIS
46is	HI_HIS
51is	HI_HIS
52is	PI_HIS
52pi	PI_PIS
53is	PI_HIS
53pi	PI_PIS

Table S4. ARGs and their corresponding antibiotic class assignments.

**ARG**	**Antibiotic class**
aad(6)	aminoglycosides
aac(6')-Ic	aminoglycosides
aac(6)-Ic	aminoglycosides
aph(3")-III	aminoglycosides
Aph(3')-Ia	aminoglycosides
aph(3")-Ia	aminoglycosides
ant(6)-Ia	aminoglycosides
aph(3")-Ib	aminoglycosides
Aph(6)-Ib	aminoglycosides
aph(6)-Id	aminoglycosides
aac(6')	aminoglycosides
aph(3')-III	aminoglycosides
aph(3')-IIIa	aminoglycosides
SRT-2	cephalosporins
patA	fluoroquinolones
patB	fluoroquinolones
pmrA	fluoroquinolones
RlmA(II)	lincosamides, macrolides
mef(A)	macrolides
msr(D)	macrolides, lincosamides, and streptogramins
erm(CX)	macrolides, lincosamides, and streptogramins
erm(F)	macrolides, lincosamides, and streptogramins
erm(B)	macrolides, lincosamides, and streptogramins
erm(X)	macrolides, lincosamides, and streptogramins
erm(A)	macrolides, lincosamides, and streptogramins
sat4	nucleoside antibiotics

Journal of Clinical Medicine

catQ	phenicols
catS	phenicols
catA16	phenicols
lsa(C)	pleuromutilins, streptogramins, lincosamides
pgpB	polymyxins
rpoB	rifamycins
tet(Q)	tetracyclines
tet(O)	tetracyclines
tet(W)	tetracyclines
tet(M)	tetracyclines
tet(B)	tetracyclines
tetB(60)	tetracyclines
tet(32)	tetracyclines
tetA(46)	tetracyclines
tetA(60)	tetracyclines
tetB(46)	tetracyclines
blaTEM-2	β-lactams
cfxA4	β-lactams
cfxA	β-lactams
blaSST-1	β-lactams
blaTEM-105	β-lactams
mrcA	β-lactams
mrcB	β-lactams
blaSPU-1	β-lactams
cfxA3	β-lactams
blaCSP-1	β-lactams
cfxA2	β-lactams
blaTEM-1B	β-lactams
blaTEM-1	β-lactams

Table S5. Pairwise PERMANOVA based on the Jaccard coefficient between study groups.

	R^2	p-value
HI_Sa *vs* PI_HIS	0.101	0.001
HI_Sa *vs* PI_PIS	0.109	0.001
HI_Sa *vs* PI_Sa	0.014	0.929
HI_Sa *vs* HI_HIS	0.159	0.001
PI_HIS *vs* PI_PIS	0.014	0.972
PI_HIS *vs* PI_Sa	0.084	0.002
PI_HIS *vs* HI_HIS	0.038	0.262
PI_PIS *vs* PI_Sa	0.081	0.001
PI_PIS *vs* HI_HIS	0.046	0.098
PI_Sa *vs* HI_HIS	0.131	0.001

Journal of Clinical Medicine

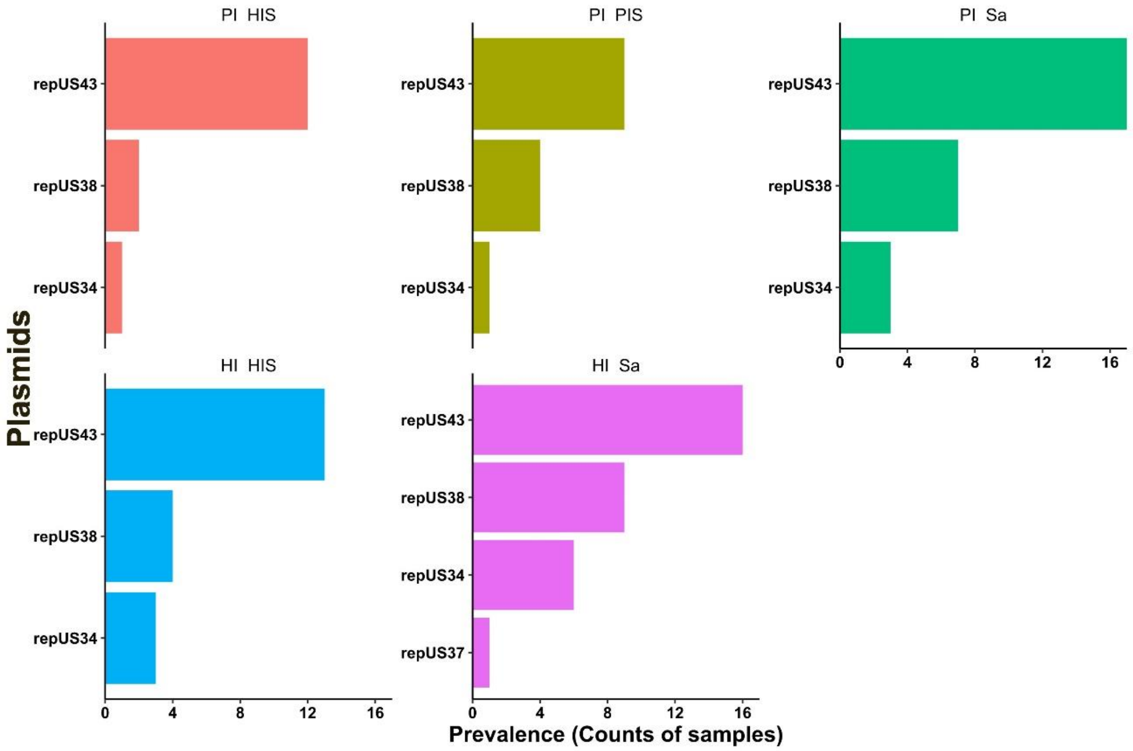

Figure S1. Prevalence of plasmids (detected using the PlasmidFinder database) across each of the five study groups (PI_HIS, PI_PIS, PI_Sa, HI_Sa and HI_HIS).

Journal of Clinical Medicine

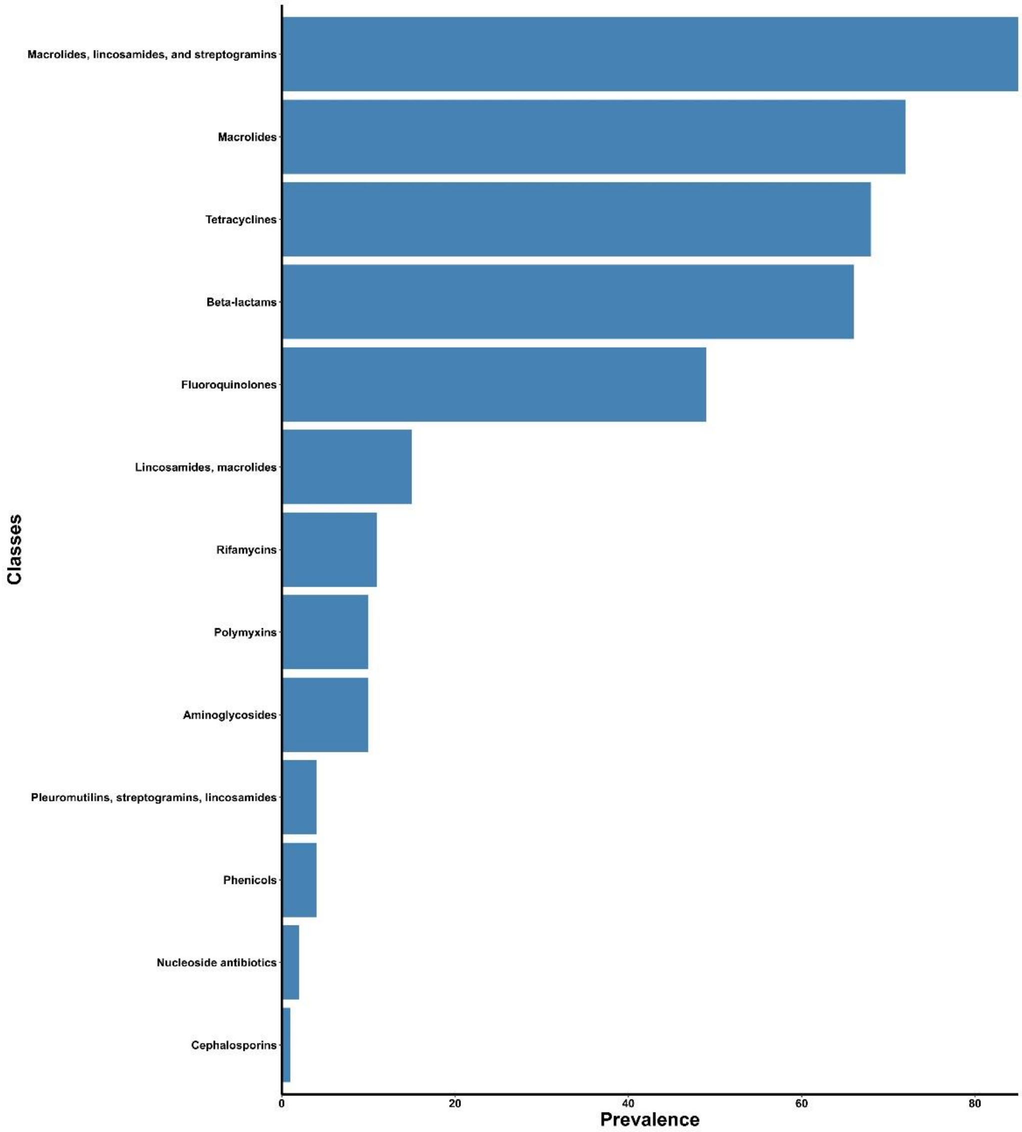

Figure S2. Prevalence of ARGs by antibiotic classes across all samples in which ARGs were detected (n = 89).

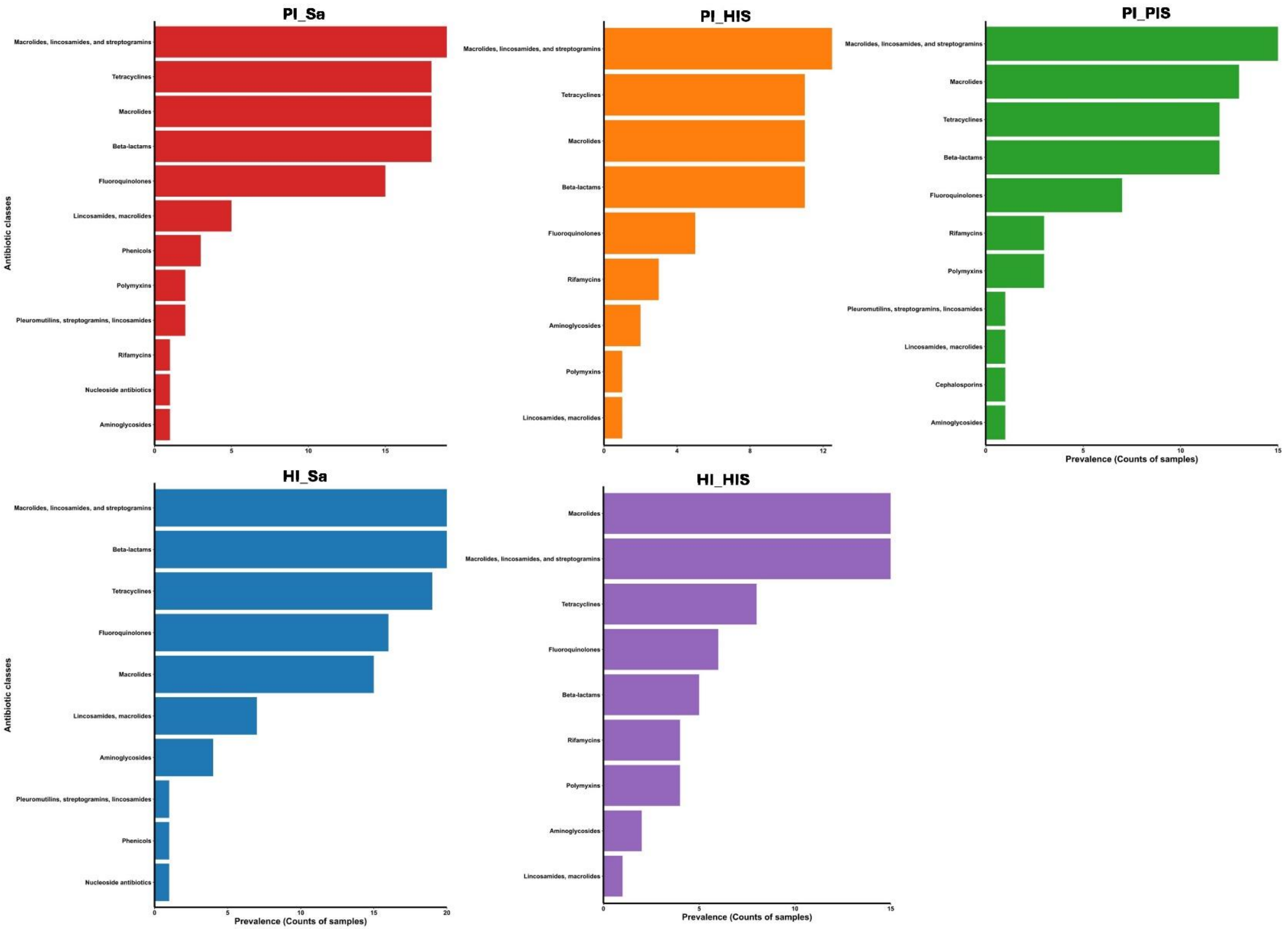

Figure S3. Prevalence of ARGs by antibiotic classes across each of the five study groups (PI_Sa, PI_HIS, PI_PIS, HI_Sa and HI_HIS).

Journal of Clinical Medicine

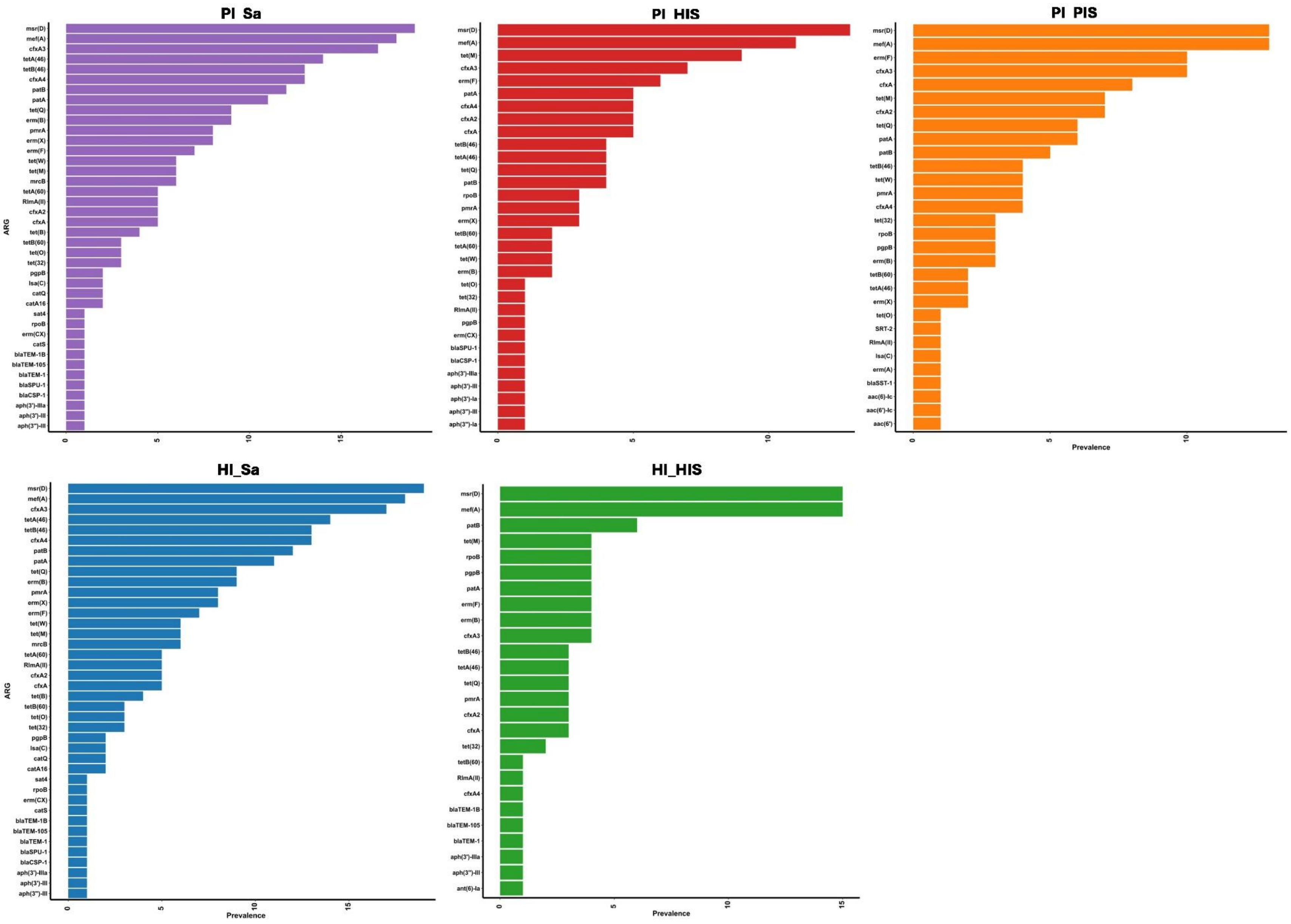

Journal of Clinical Medicine

MDPI

Figure S4. Prevalence of ARGs across each of the five study groups (PI_Sa, PI_HIS, PI_PIS, HI_Sa and HI_HIS).

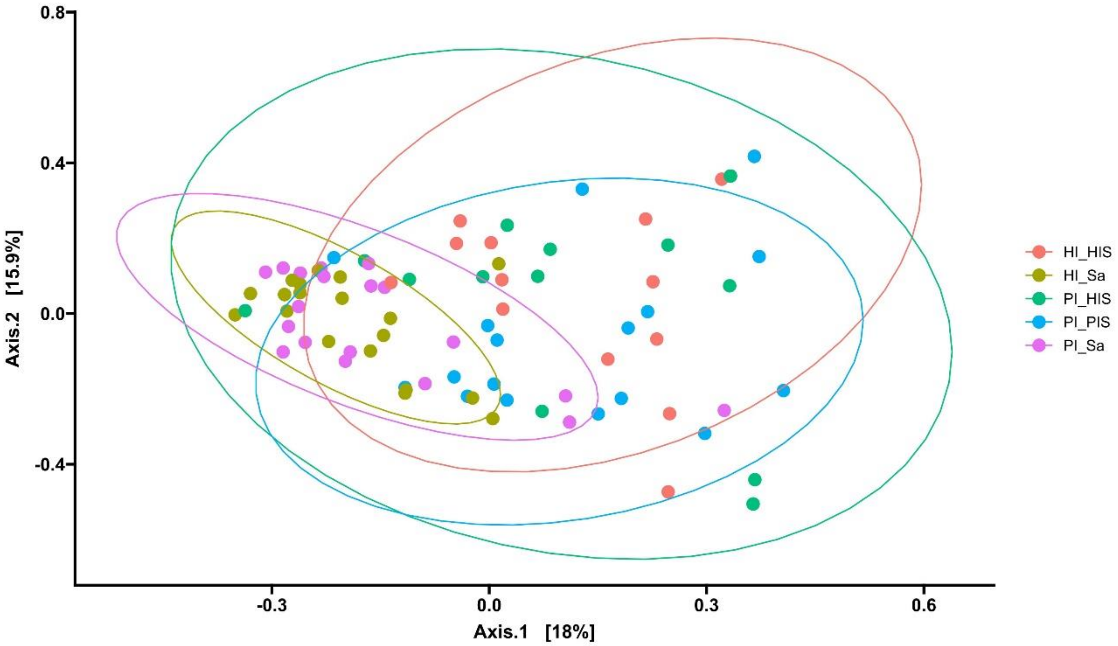

Figure S5. PCoA of Jaccard distance showing the dissimilarity of the ARG profiles between groups. PERMANOVA: $R^2 = 0.122$, $p = 0.001$.
